# Synthesis of Poly(δ-valerolactone)-poly(*N,N*-dimethylacrylamide) Supermicelles in Concentrated Aqueous Media via Reverse Sequence Polymerization-Induced Self-Assembly

**DOI:** 10.1021/acs.macromol.6c00704

**Published:** 2026-04-03

**Authors:** Matthew A. H. Farmer, Oleksandr O. Mykhaylyk, Osama M. Musa, Steven P. Armes

**Affiliations:** † School of Mathematics and Physical Sciences, 7315 University of Sheffield, Dainton Building, Brook Hill, Sheffield S3 7HF, U.K.; ‡ Ashland Specialty Ingredients, 1005 US 202/206, Bridgewater, New Jersey 08807, United States

## Abstract

Highly anisotropic diblock copolymer nanoparticles with poly­(δ-valerolactone) (PVL) cores are prepared via reverse sequence polymerization-induced self-assembly (PISA). A hydrophilic monomer, *N,N*-dimethylacrylamide (DMAC), is combined with a trithiocarbonate-capped PVL precursor and polymerized via reversible addition–fragmentation chain transfer (RAFT) polymerization. This synthesis is initially conducted in the bulk; however, the reaction mixture is subsequently diluted with preheated deoxygenated water once a suitable intermediate DMAC conversion (37–42%) has been attained. Full DMAC conversion was achieved when targeting PVL–PDMAC diblock copolymer nanoparticles at 30% w/w solids, with a high chain extension efficiency and good control (*M*_w_/*M*_n_ ≤ 1.29) being achieved for this RAFT polymerization. The initial copolymer morphology is ill-defined; however, depending on the target PVL–PDMAC composition, either anisotropic semiflexible rod-like particles or so-called supermicelles can be formed on aging such PVL–PDMAC diblock copolymer dispersions for up to 19 weeks at 20 °C. This is attributed to slow crystallization of the PVL block, which is initially amorphous immediately after synthesis, as judged by X-ray diffraction studies, but gradually becomes more semicrystalline on aging. Thus, the evolution in copolymer morphology is driven by crystallization-driven self-assembly (CDSA). The aged nanoparticles were characterized by transmission electron microscopy and small-angle X-ray scattering. The semiflexible rods were also subjected to shear-induced polarized light imaging analysis, with shear-induced alignment remaining for up to 1 h after cessation of shear.

## Graphical abstract

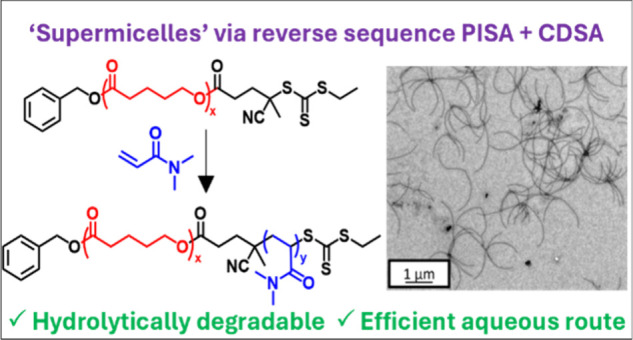

## Introduction

Synthetic vinyl polymers are remarkably diverse materials that have become both ubiquitous and indispensable in the modern world.^1–4^ However, such materials typically have poor (bio)­degradability profiles, which leads to their long-term persistence in the environment for many years after their initial use.^1,5,6^ Indeed, anthropologists have suggested that plastic waste can be used as a geological indicator for a new Anthropocene era.^7^ Moreover, the global production of plastic waste is predicted to grow significantly over the next few decades.^1,8^ Hence, it is essential to focus on the design of next-generation synthetic polymers that exhibit tunable degradability.

Microscopic polymer particles are widely used in paints, coatings, lubricants, agrochemical and cosmetic formulations.^9–12^ These particles are typically prepared using vinyl monomers, which produce nondegradable polymer chains.^9–12^ Thus, such particles are likely to have a negative long-term environmental impact.^13–18^ For example, polystyrene particles of 50–180 nm diameter can penetrate the blood–brain barrier of Crucian carp, which may cause behavioral disorders.^18^ Similarly, the uptake of 30 nm polystyrene nanoparticles by blue mussels can also result in irregular behavior, e.g., a reduction in filtering activity for such bivalve organisms.^13^

Polymerization-induced self-assembly (PISA) is a versatile platform technology that enables the efficient synthesis of block copolymer nanoparticles directly in the form of concentrated colloidal dispersions at up to 50% w/w solids.^19–24^ Depending on the target diblock copolymer composition and the reaction conditions, spherical nanoparticles,^25–31^ worms,^32–34^ or vesicles^26,27,33^ can be prepared and reversible addition–fragmentation chain transfer (RAFT) polymerization is most commonly used for such syntheses.^35^ Regardless of the precise nature of the polymerization, PISA involves chain extension of a soluble homopolymer precursor using a suitable vinyl monomer that results in the formation of an insoluble second block at a critical degree of polymerization (DP).^36,37^ This approach is suitable for many types of vinyl monomers, works for both aqueous and nonaqueous formulations, and is amenable to scale-up.^25,38–46^ However, the resulting nanoparticles are typically nondegradable. Recently, there has been a concerted synthetic effort to develop PISA protocols that incorporate cleavable bonds into the copolymer chains to confer degradability.^47–50^

It is well-known that cyclic ketal acetals (CKAs), such as 2-methylene-4-phenyl-1,3-dioxolane (MPDL) and 5,6-benzo-2-methylene-1,3-dioxepane (BMDO), α-lipoic acid, or thionolactones, such as dibenzo­[c,e]­oxepane-5-thione (DOT), can be statistically copolymerized with vinyl monomers via radical ring-opening polymerization (rROP) to incorporate cleavable ester, disulfide, or thioester bonds within otherwise nondegradable polymer chains.^51–57^ Recently, Nicolas and co-workers have combined rROP with PISA,^58–63^ resulting in spherical or vesicular nanoparticles bearing cleavable ester bonds in the core and/or corona blocks. However, cyclic comonomers, such as CKAs and DOT, require multistep syntheses that are not particularly efficient.

Recently, we reported that *reverse sequence* aqueous PISA enables the efficient synthesis of core-degradable nanoparticles in the form of concentrated aqueous dispersions.^64–66^ This new approach involves using a hydrophobic aliphatic polyester precursor [e.g., poly­(ε-caprolactone) or poly­(*L*-lactide)] functionalized with a suitable RAFT end-group to polymerize *N,N*-dimethylacrylamide (DMAC), starting either in the bulk or in 80% w/w aqueous solution. Once sufficient DMAC had been polymerized, preheated degassed water was added to the reaction mixture at a predetermined time point. In contrast to traditional PISA syntheses, where nucleation occurs when the growing second block becomes insoluble at a certain critical DP,^26,27^ nucleation during *reverse sequence* PISA occurs immediately after water addition. A transient lamellar phase is initially formed, which rapidly reorganizes to produce nascent spherical nanoparticles.^67^ Thereafter, the mean diameter of the polyester-core spheres is *reduced* as the remaining DMAC monomer is consumed and the PDMAC stabilizer chains grow longer since this leads to a lower aggregation number.^67^ Recently, a similar *reverse sequence* PISA protocol was utilized by Couturaud and co-workers to prepare poly­[(ethylene carbonate)-*co*-(ethylene oxide)]-PDMAC spherical nanoparticles.^68^

The maximum amount of CKA per copolymer chain reported in the literature is 11% by mass,^59^ while around 10% by mass has been achieved in the case of DOT.^61^ Such limitations are due to the unfavorable comonomer reactivity ratios between CKAs and methacrylic monomers^59^ (plus the hydrophobic nature of DOT^60,61^ ). This is sufficient to produce oligomers via hydrolytic degradation but not small molecules.^58–63^ In contrast, the proportion of the hydrolytically degradable poly­(ε-caprolactone) block in *reverse sequence* aqueous PISA formulations can be up to 36% by mass,^64^ which is substantially higher than that achieved by statistical copolymerization of CKA or DOT. On the other hand, only the core-forming block is degradable using the former approach, whereas introducing either a CKA or DOT comonomer can confer degradability on the steric stabilizer block as well. Herein, we expand the scope of this new strategy to introduce poly­(δ-valerolactone) (PVL) as an alternative aliphatic polyester precursor; see Figure 1.

**Figure 1:**
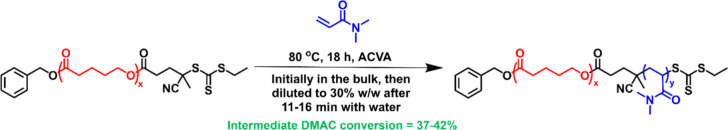
Synthesis of PVL_*x*_-PDMAC_*y*_ nanoparticles prepared at 80 °C via reverse sequence aqueous PISA. Initially, the RAFT polymerization of DMAC is conducted in the bulk, with subsequent dilution to target 30% (w/w) solids using preheated deoxygenated deionized water, which was added at an intermediate DMAC conversion of 37–42%. Conditions: [PVL_*x*_-TTC]/[ACVA] molar ratio = 5.0; target PDMAC DP = 30 or 47.

## Results and Discussion

Two monohydroxy-capped PVL precursors were prepared via ring-opening polymerization (ROP) of δ-valerolactone using benzyl alcohol as an initiator according to a literature protocol.^69^ ^1^H NMR spectroscopy and gel permeation chromatography (GPC) were used to assess the mean DP of each PVL–OH precursor and molecular weight distribution, respectively (see Figures 2, S1, and S2). One precursor was determined to have a DP of 24 (*M*_n_ = 3.7 kg mol^–1^; *M*_w_/*M*_n_ = 1.13) and the other had a DP of 36 (*M*_n_ = 5.6 kg mol^–1^; *M*_w_/*M*_n_ = 1.30). These precursors are denoted as PVL_24_–OH and PVL_36_–OH, respectively.

**Figure 2:**
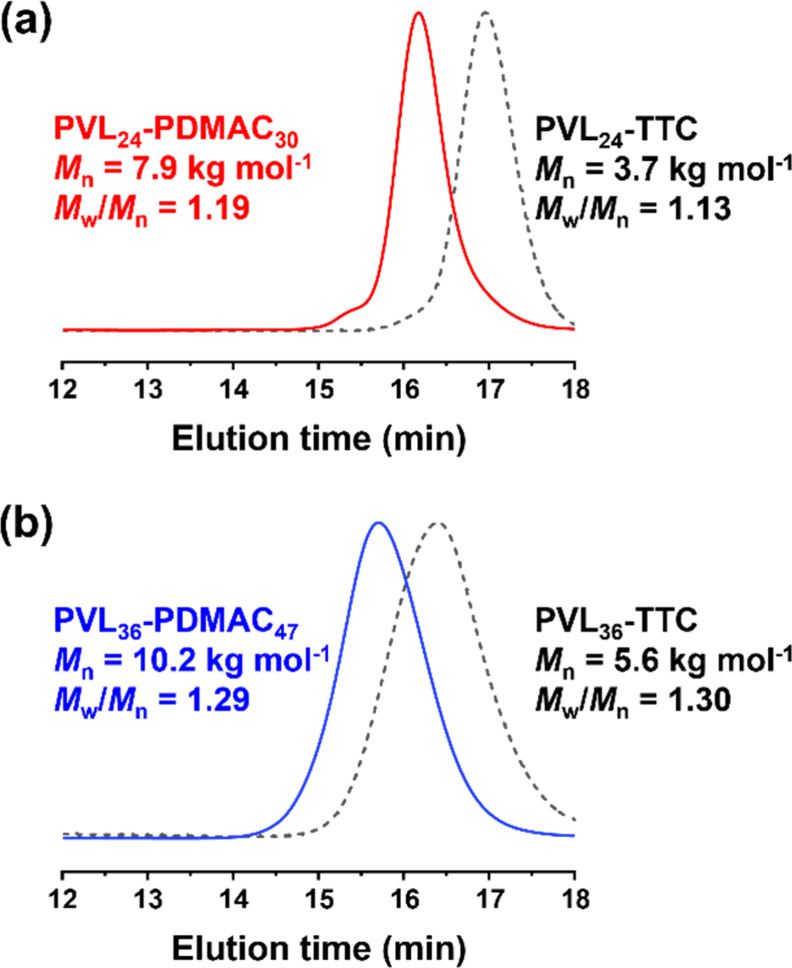
DMF GPC curves (refractive index detector) recorded for (a) the PVL_24_-PDMAC_30_ diblock copolymer and its corresponding PVL_24_-TTC precursor and (b) the PVL_36_-PDMAC_47_ diblock copolymer and its corresponding PVL_36_-TTC precursor.

To conduct the *reverse sequence* aqueous PISA synthesis of PVL–PDMAC nanoparticles, each precursor was first esterified with a carboxylic-acid-functionalized trithiocarbonate (CEPA) to produce the desired PVL_*x*_-TTC. ^1^H NMR spectroscopy was used to assess the mean degree of esterification (see Figures S3 and S4). Comparison of the integrated proton signals at 1.91 ppm assigned to the methyl group of the RAFT agent with that of the unique PVL backbone signals at 4.07 ppm indicated a mean degree of esterification of approximately 100% for each precursor. Moreover, UV GPC studies confirmed that no residual CEPA RAFT agent was present (Figure S5). DSC analysis indicated that PVL_24_-TTC and PVL_36_-TTC were both semicrystalline, exhibiting a melting temperature (*T*_m_) of 46 and 50 °C, respectively (see Figure S6).

Each PVL_*x*_-TTC precursor was then mixed in turn with a DMAC monomer, which was polymerized in the bulk at 80 °C. A PDMAC DP of 30 was targeted when using the PVL_24_-TTC precursor, and a PDMAC DP of 47 was targeted when using the PVL_36_-TTC precursor. During DMAC polymerization, a substantial increase in viscosity was observed within 11–16 min, which corresponded to an intermediate DMAC conversion of 37–42%. At this time point, additional preheated degassed water was added to target 30% (w/w) solids at full DMAC conversion. [N.B. Such water addition required precise control in terms of timing. Macroscopic precipitation occurred if water was added prior to the formation of a more viscous reaction milieu. On the other hand, if water addition was delayed, the reaction mixture solidified and could not be diluted.] DMF GPC analysis of the resulting PVL_24_-PDMAC_30_ and PVL_36_-PDMAC_47_ diblock copolymers indicated high chain extension efficiencies and relatively narrow molecular weight distributions (*M*_w_/*M*_n_ < 1.30, see Figure 2a,b), while ^1^H NMR spectroscopy studies indicated that essentially full DMAC conversion was achieved in each case (see Figure 3). Such formulations provide convenient access to diblock copolymer nanoparticles comprising up to 46% of the hydrolytically degradable PVL component by mass. In preliminary studies, reverse sequence PISA syntheses were also attempted using a PVL_98_ precursor (*M*_n_ = 10.2 kg mol^–1^). However, the DMAC monomer was unable to solubilize this higher molecular weight precursor, and only an inhomogeneous reaction mixture could be obtained (data not shown).

**Figure 3:**
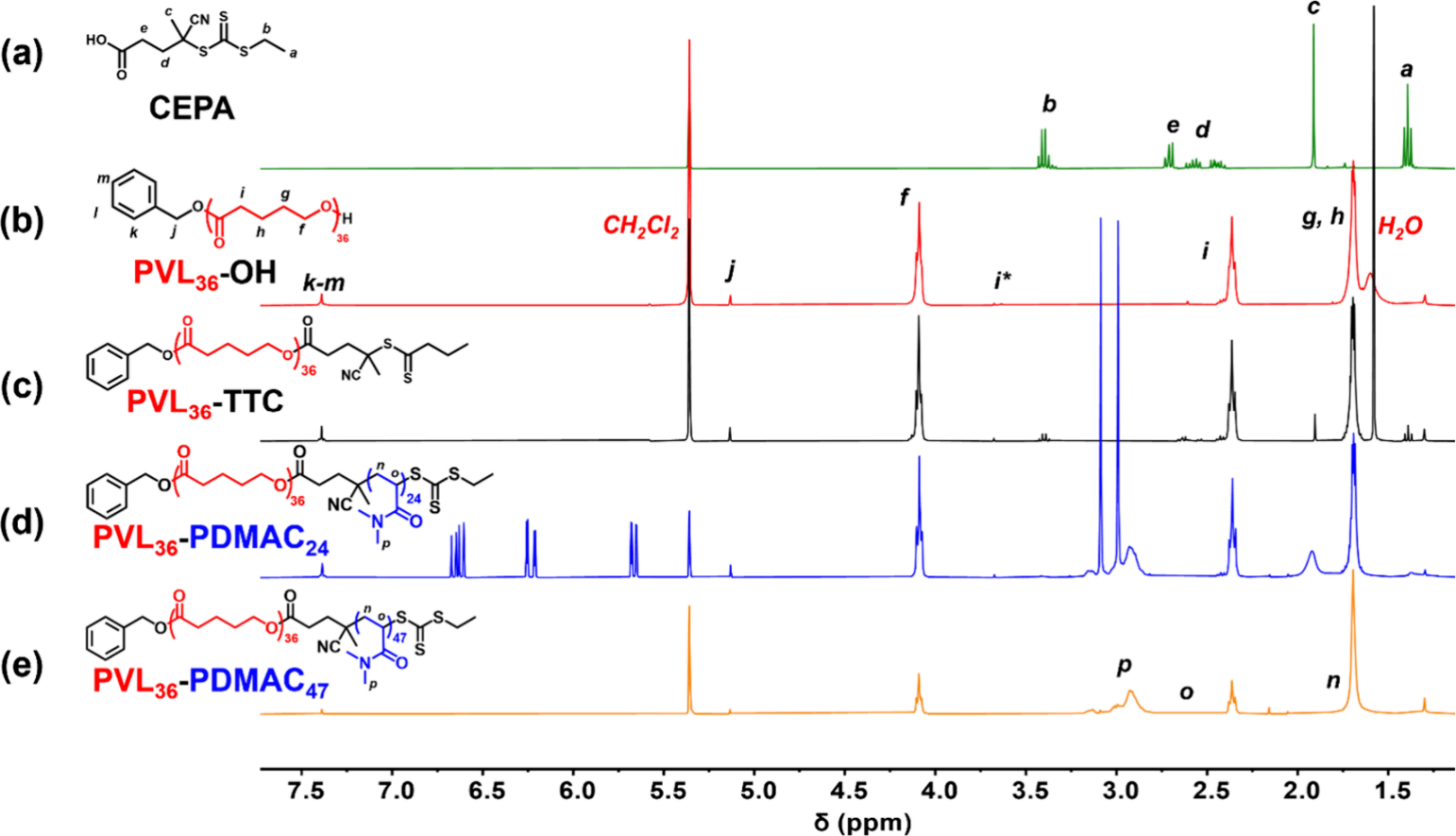
^1^H NMR spectra (CD_2_Cl_2_) recorded for (a) the carboxylic acid-functionalized RAFT agent (CEPA), (b) the PVL_36_–OH precursor (where i* represents the terminal *h* protons), (c) the PVL_36_-TTC precursor, (d) PVL_36_-PDMAC_24_ (51% DMAC conversion—note the three vinyl signals between 5.5 and 6.7 ppm assigned to the unreacted DMAC monomer), and (e) PVL_36_-PDMAC_47_ (>99% DMAC conversion).

Our previous protocol for reverse sequence aqueous PISA utilized a trithiocarbonate-capped poly­(ε-caprolactone) (PCL) precursor and yielded spherical nanoparticles.^64^ At first glance, this may seem surprising because PCL is a semicrystalline polymer and it is well-known to undergo crystallization-driven self-assembly (CDSA) to produce rod-like nanoparticles.^70,71^ However, we have recently shown that the formation of spherical nanoparticles is simply because the reaction temperature of 80 °C exceeds the Tm of 45 °C for PCL.^67^ Thus, the PCL cores of the resulting PCL–PDMAC nanoparticles remain amorphous throughout the DMAC polymerization. Furthermore, the minimum nucleus size to form a stable PCL crystal was calculated to be around 15 nm, which is larger than the core diameter of the spherical PCL–PDMAC nanoparticles (8.8 nm).^67^ Hence, it is clear that nanoconfinement of the PCL cores prevents their crystallization on cooling to ambient temperature.^67^

Compared with the corresponding PCL–PDMAC syntheses, significantly lower PDMAC DPs can be targeted when preparing PVL–PDMAC copolymers. Thus, the initial PVL–PDMAC nanoparticles formed at 80 °C should have a larger mean core diameter according to the well-established theory of block copolymer self-assembly.^72^ Importantly, if these PVL cores are larger than the critical size of nuclei required for crystallization, this should allow their (slow) crystallization after cooling from 80 to 20 °C. In principle, this should lead to a gradual evolution in the copolymer morphology via CDSA.

The PVL_24_-TTC precursor was studied by SAXS. Analysis of its scattering pattern using the well-known equation^74^ *q* = 2π/*d* indicated a mean lamellar stacking periodicity of 8.8 nm, see Figure 4. This is comparable to the lamellar stacking of 7.3 nm reported for a PVL_146_ homopolymer by Furuhashi et al.^73^ This confirms the semicrystalline nature of the PVL_24_-TTC precursor despite its relatively low DP.

**Figure 4:**
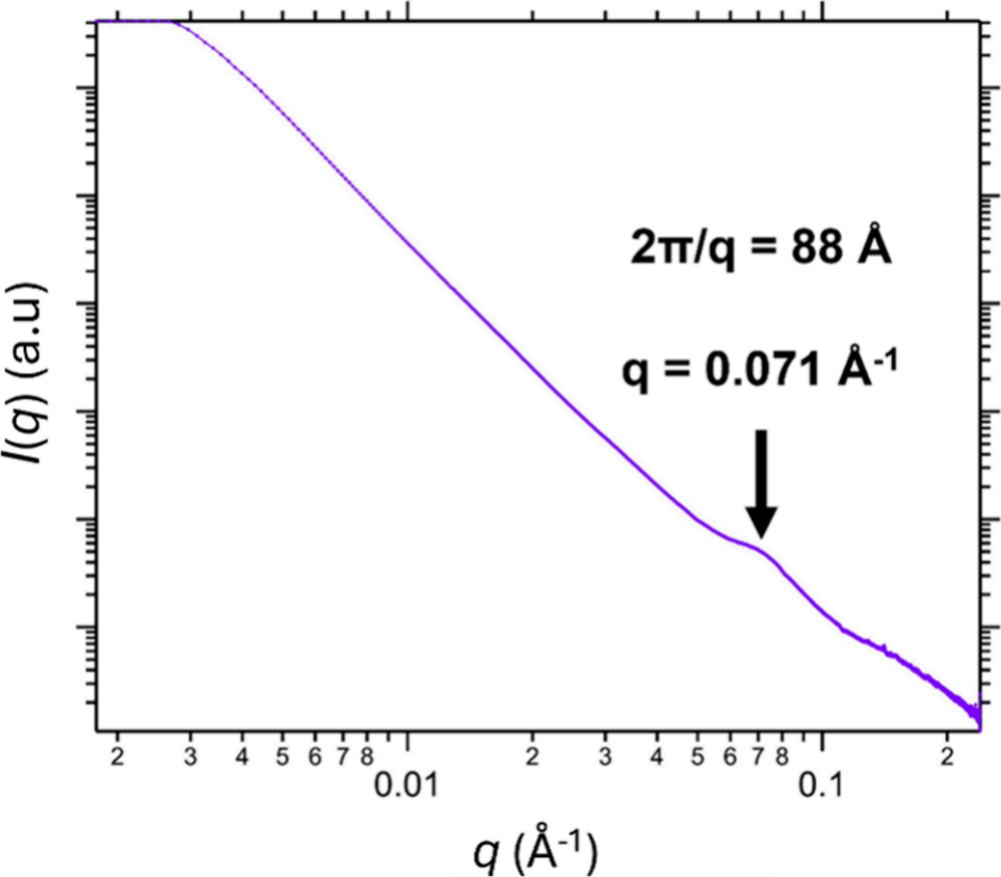
SAXS pattern recorded for the solid PVL_24_-TTC precursor used to prepare the PVL_24_-PDMAC_30_ nanoparticles reported in this study. The structure peak at *q* ∼ 0.071 Å^–1^ corresponds to a lamellar period, *d*, of 88 Å or 8.8 nm (as calculated using the well-known relation, *q* = 2π/*d*).^74^

TEM analysis of the PVL_24_-PDMAC_30_ formulation indicated that no well-defined nanoparticles were visible immediately after synthesis (see Figure 5a and Scheme S1) or after aging for 1 week at 20 °C (see Figure 5b and Scheme S1). However, after aging at 20 °C, some rod-like nanoparticles begin to form (see Figure 5c). Such particles grow in size over time: after 16 weeks of aging at 20 °C, semiflexible rods were visible as a pure phase (see Figure 5d,e). However, no further change in the nanoparticle morphology was discernible after a further 3 weeks of aging at 20 °C (see Figure 5f).

**Figure 5:**
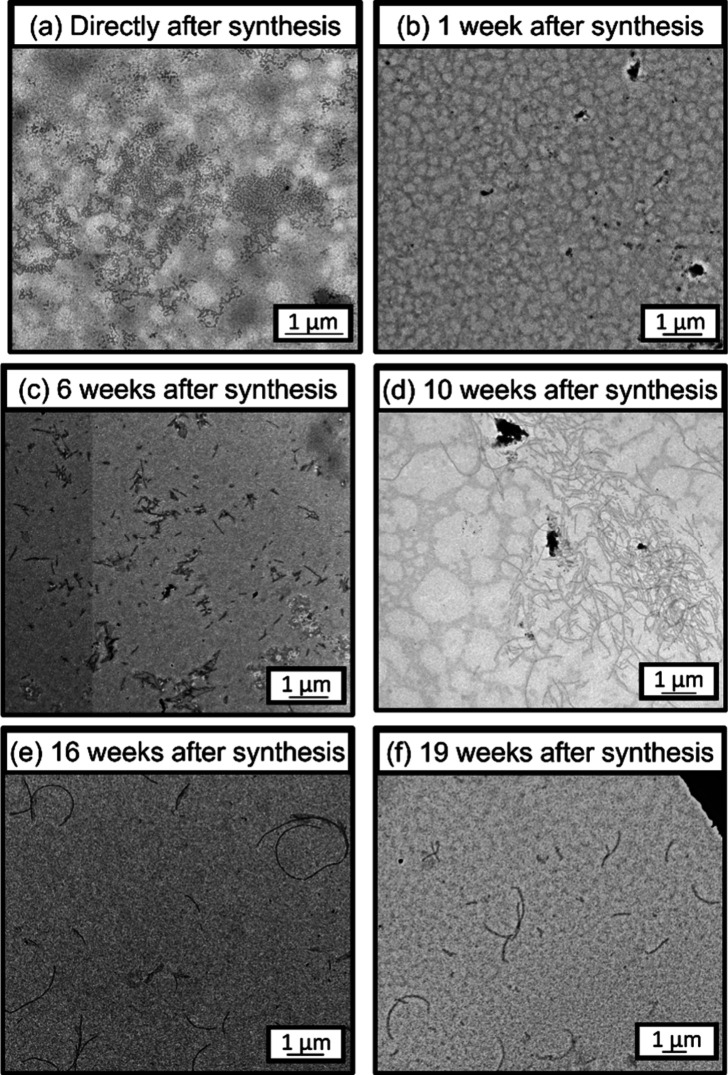
Representative TEM images recorded for a 0.1% w/w aqueous dispersion of PVL_24_-PDMAC_30_ nanoparticles recorded (a) immediately after synthesis and after aging at 20 °C for (b) 1, (c) 6, (d) 10, (e) 16, and (f) 19 weeks.

The PVL_36_-PDMAC_47_ formulation produced highly anisotropic worm-like aggregates immediately after synthesis (see Figure 6a). The mean width of the initial worms is estimated to be 33 ± 5 nm (based on the digital image analysis of at least 50 nanoparticles in each case). Such worm-like nanoparticles are well-known for various aqueous PISA formulations and are characteristic of conventional self-assembly behavior, rather than CDSA.^19,20,23,75^ Thus, the core-forming PVL_36_ block appears to be amorphous immediately after self-assembly.

**Figure 6:**
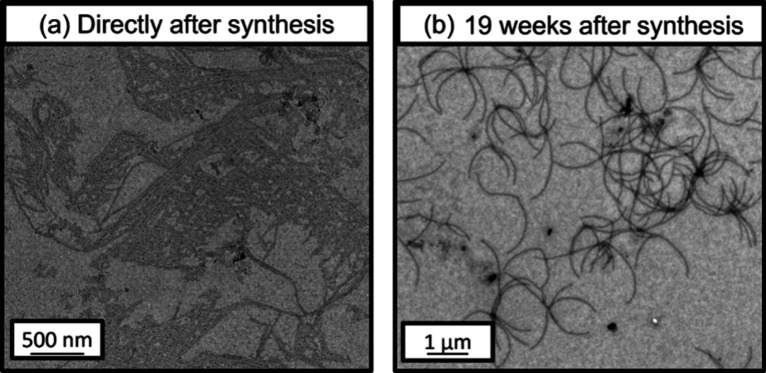
Representative TEM images recorded for a 0.1% w/w aqueous dispersion of PVL_36_-PDMAC_47_ nanoparticles recorded (a) immediately after synthesis and (b) after aging for 19 weeks at 20 °C.

However, aging this 30% w/w aqueous dispersion for 19 weeks at 20 °C results in a remarkable evolution in copolymer morphology to produce so-called ‘supermicelles’ (see Figure 6b). Aging studies were also conducted at 30 °C in an attempt to reduce the time scale required for supermicelle formation. However, only rods were observed in such experiments (data not shown). A similar ‘supermicelles’ morphology has been reported by the groups of Winnik and Manners,^76–79^ who used poly­(ferrocenyldimethylsilane) (PFS) as a core-forming block when conducting traditional CDSA processing using binary alkane/alcohol mixtures. However, their strategy required the addition of individual PFS-based diblock copolymer chains to a seeded dispersion of initial nanoparticles rather than a one-pot synthesis. Moreover, ‘supermicelles’ were obtained at a copolymer concentration of only 2% w/w, as opposed to a 30% w/w aqueous dispersion.^79^ Furthermore, the structure-directing PFS block is not hydrolytically degradable, unlike the PVL block employed in the present study.

Clearly, the initial copolymer morphology evolves over time. This is likely to be the result of CDSA driven by crystallization of the PVL core-forming block. To examine this possibility, we conducted XRD studies. Sharp diffraction peaks corresponding to the {110} and {200} lattice planes of the δ-PVL orthorhombic unit cell^73^ at 2θ = 22° and 24°, respectively, were observed in XRD patterns recorded for the solid PVL-TTC precursors (see Figure 7a,b).^80^ When targeting PVL_24_-PDMAC_30_ or PVL_36_-PDMAC_47_, no crystallinity was detected via XRD immediately after synthesis (see Figure 7a,b). However, aging an aqueous dispersion of PVL_24_-PDMAC_30_ nanoparticles for 1 week at 20 °C led to an increase in mean degree of crystallinity, *D*_c_, from 0% to 12% (see Figure 7a). After aging for an additional 2 weeks at 20 °C, the *D*_c_ increased up to 18%. Unexpectedly, further aging for 19 weeks resulted in a *reduction* in *D*_c_ to 11%. GPC analysis of the fresh and aged copolymer chains indicates partial hydrolytic degradation of the PVL block over this time frame, which may account for this lower crystallinity (see Figure S7).

**Figure 7:**
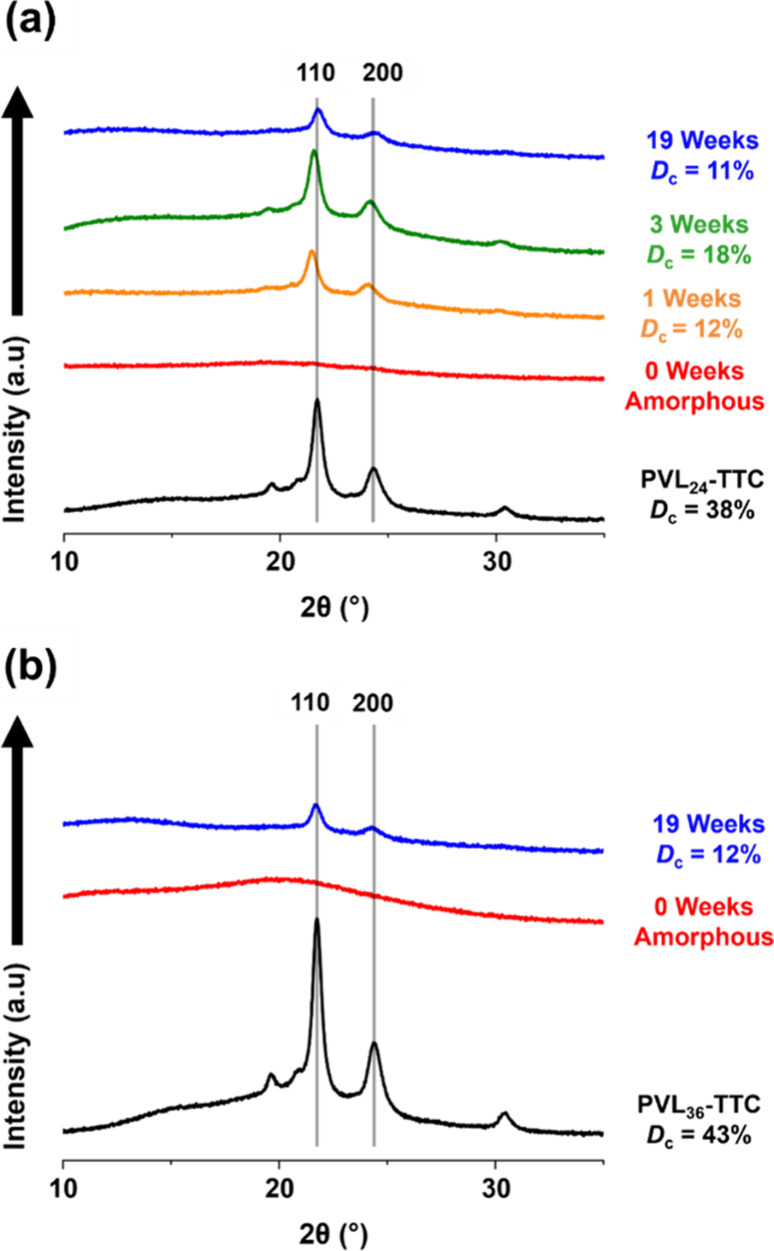
XRD patterns recorded for (a) the PVL_24_-TTC precursor (black curve) and the corresponding freeze-dried aqueous dispersion of PVL_24_-PDMAC_30_ nanoparticles obtained directly after synthesis (red curve), after 1 week aging at 20 °C (orange curve), after 3 weeks aging at 20 °C (green curve), and after 19 weeks aging at 20 °C (blue curve). (b) XRD patterns recorded for the PVL_36_-TTC precursor (black curve) and the corresponding freeze-dried aqueous dispersion of PVL_36_-PDMAC_47_ nanoparticles obtained either directly after synthesis (red curve) or after 19 weeks of aging at 20 °C (blue curve).

A comparable increase in *D*_c_ from 0 to 12% was also observed after aging PVL_36_-PDMAC_47_ nanoparticles to form ‘supermicelles’ after storage for 19 weeks at 20 °C (see Figure 7b). One reviewer of this paper asked (i) whether chain mobility affects the rate of crystallization of the core-forming PVL block and (ii) whether interparticle fusion plays an important role in this regard. We do not know the answers to these intriguing questions, which are included here, with the aim of inspiring further studies.

The morphology of the ‘supermicelles,’ formed by core-connected flexible cylindrical arms (see the TEM image in Figure 6b), resembles that of a star polymer. Hence, a star polymer scattering model that assumes semiflexible arms^81^ can be employed for analysis of the SAXS pattern shown in Figure 8, albeit with some modification to account for the cross-section of the ‘supermicelle’ arms.

**Figure 8:**
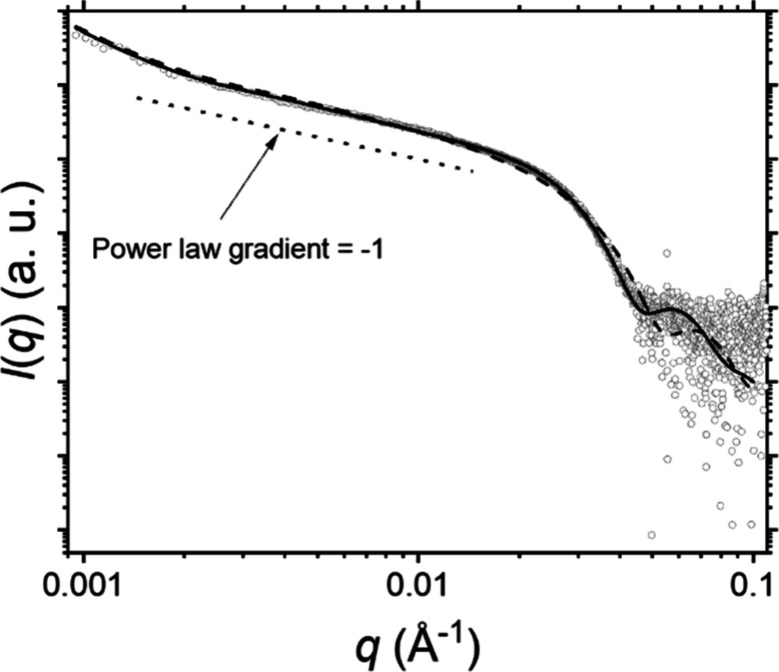
SAXS pattern recorded for a 1.0% w/w aqueous dispersion of PVL_36_-PDMAC_47_ “supermicelles” (gray open circle symbols) with a corresponding fit using a modified star polymer scattering model either with a structure factor (black solid curve) or with no structure factor (black dashed curve). The dotted line indicates a power law gradient of – 1 as a reference.

For simplicity, to a first approximation, the core and corona regions of the arms are both assumed to have an average homogeneous cross-section corresponding to that of a star comprising core-connected semiflexible rods. TEM images indicate a relatively high aspect ratio for the arms (Figure 6b). Thus, using an approximation that is commonly applied to long cylinders, i.e., that their length is significantly larger than their cross-sectional radius,^82^ the scattering equation for ‘supermicelles’ comprising semiflexible arms can be expressed as the product of two separate functions representing a longitudinal term and a cross-sectional term:

$$l(q)=K.P_{\mathrm{ps}}(q,L,b,f)\int_{0}^{\infty }P_{\mathrm{cs}}(q,r)\Psi (r)dr$$ 1

where *K* is the scaling coefficient related to the scattering length density contrast of the ‘supermicelles’, their volume fraction, as well as the particle volume. *P*_ps_ (*q*, *L*, *b*, *f*) is the star polymer form factor, where *L* is the average contour length of a single arm, *b* is its Kuhn length, and *f* is the mean number of arms per ‘supermicelle’. This form factor has been derived elsewhere^81^ and is incorporated in the SASfit software package.^83^ Since scattering features associated with long arms exceeding 1 μm (Figure 6b) are not resolved in the scattering experiment for *q* < 0.001 Å^–1^ (Figure 8), the arm contour length was fixed at 1 μm in the SAXS analysis. Assuming a homogeneous SLD across the arms, the cross-sectional term for the ‘supermicelles’ is given by

$$P_{\mathrm{cs}}\left(q,r\right)=[2J_{1}\left(qr\right)/\left(qr\right){]}^{2}$$ 2

where *J*_1_(*qr*) is the first-order Bessel function of the first kind and *r* denotes the effective cross-sectional radius of the arms. Equation 1 also accounts for the dispersity of the arm cross-section radius. A Gaussian distribution was used for the data fitting, see eq 3, where *R* is the mean cross-sectional radius of the arm and σ_*R*_ is its standard deviation.

$$\Psi (r)=\frac{1}{\sqrt{2\pi \sigma_{R}^{2}}}e^{-(r-R{)}^{2}/2\sigma_{R}^{2}}$$ 3

This modified star polymer scattering model was implemented within the SASfit software package^83^ using the plugin tool and applied to analyze the SAXS pattern. The derived model for ‘supermicelles’ with semiflexible arms (eq 1) produced a reasonable fit in the low *q* region (Figure 8, dashed curve), yielding an arm Kuhn length *b* of approximately 250 nm and indicating an average of 4–5 arms per ‘supermicelle’, which is consistent with TEM observations (Figure 6b). However, there were discernible deviations at intermediate *q* associated with the ‘supermicelle’ cross-section scattering. Such deviations are likely to be related to a structure factor component owing to interarm interactions. Interactions between worm-like micelles are commonly accounted for using the Polymer Reference Interaction Site Model (PRISM).^84^ However, incorporation of a PRISM-based structure factor into the scattering equation did not account for the observed deviation. This is likely because PRISM is derived for systems of randomly oriented, interacting worm-like micelles, whereas the ‘supermicelles’ studied herein comprise core-connected arms. In contrast, inclusion of a hard-sphere structure factor with the Percus–Yevick closure [*S*_PY_(*q*, *R*_sf_, *n*_sf_), where η_sf_ is the effective volume fraction of the interacting arms and 2*R*_sf_ is the mean distance between centers for two interacting arms], implemented using a monodisperse approximation, see eq 4, yielded a good fit over the entire *q* range (Figure 8, solid curve).

$$l\left(q\right)=K.S_{\mathrm{PY}}\left(q,R_{\mathrm{sf}},n_{\mathrm{sf}}\right)P_{\mathrm{ps}}\left(q,L,b,f\right)\int_{0}^{\infty }P_{\mathrm{cs}}\left(q,r\right)\Psi \left(r\right)dr$$ 4

This structure factor is considered more appropriate for describing interactions between ‘supermicelle’ arms, which are topologically connected and may be spatially correlated over relatively long distances. Model fitting indicates that the effective diameter of the arms, 2*R* ± 2σ_*R*_, is 16.2 ± 2.4 nm. The interarm distance of 2*R*_sf_ = 19.8 nm, obtained from structure factor parameters (*R*_sf_ = 9.9 nm and η_sf_ = 0.08), was also consistent with this result.

Shear-induced polarized light imaging (SIPLI) was also used to confirm the anisotropic nature of the PVL_24_-PDMAC_30_ semiflexible rods.^85–87^ In SIPLI experiments, anisotropic nanoparticles undergo alignment under applied shear to diffract polarized light and produce a characteristic Maltese cross pattern.^86^ Subjecting a 30% w/w aqueous dispersion of PVL_24_-PDMAC_30_ nanoparticles (prepared after aging this formulation for 19 weeks at 20 °C) to shear at either 1 or 10 s^–1^ produced a faint Maltese cross, see Figure S8. However, this distinctive motif is much more prominent at an applied maximum shear rate of 100 s^–1^, see Figure 9a. Remarkably, this Maltese cross was retained for 1 h after shear cessation, indicating that the semiflexible rods remained aligned for prolonged periods of time (see Figure 9b).

**Figure 9:**
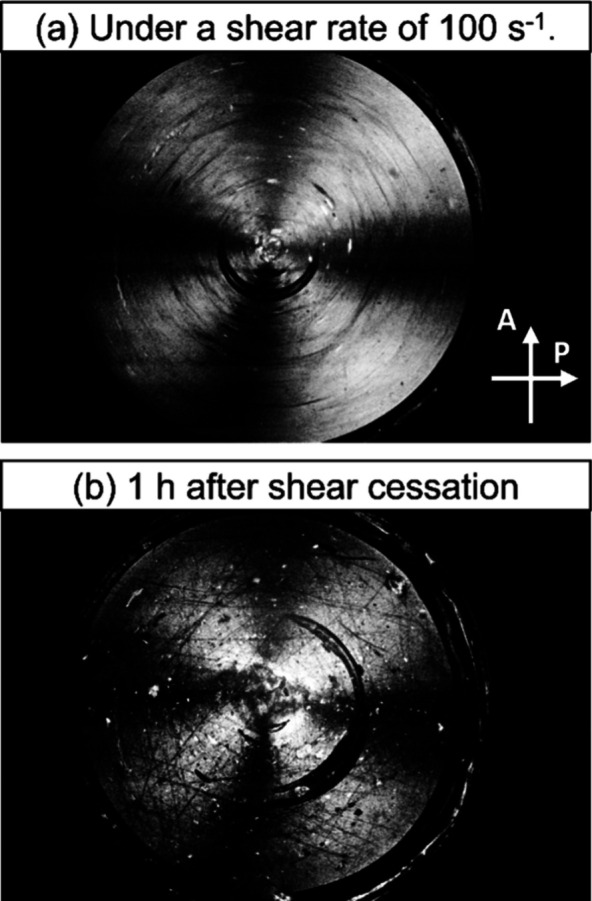
Polarized light images recorded at 20 °C for a 30% w/w aqueous dispersion of PVL_24_-PDMAC_30_ nanoparticles (a) under an applied maximum shear rate of 100 s^–1^ and (b) 1 h after contraction of the shear flow. The distinctive Maltese cross indicates shear alignment of the semiflexible rods under such conditions. The original image was converted into grayscale for clarity. Orientation of analyzer and polarizer used for taking the images are shown by white arrows.

When similar SIPLI studies were performed on a 20% w/w aqueous dispersion of diblock copolymer worms, a Maltese cross was observed at an applied shear of 1 s^–1^ but this feature disappeared within 8.3 min after cessation of shear.^86^

## Conclusions

Our *reverse sequence* PISA protocol has been extended to include a third hydrolytically degradable aliphatic polyester precursor, PVL. For chain extension with DMAC, the PVL content of the resulting PVL–PDMAC diblock copolymer can be up to 45% by mass. This is substantially higher than that achieved for aqueous PISA syntheses involving the radical ring-opening copolymerization of either CKAs or thionolactones (10–11% by mass).^56,58^ It is also significantly higher than that achieved for PCL–PDMAC nanoparticles prepared via *reverse sequence* aqueous PISA.^64,65^

According to SAXS analysis, the lamellar periodicity observed for the semicrystalline PVL homopolymer is 8.8 nm, while DSC studies indicate a *T*_m_ of 46–50 °C depending on its mean DP. The PVL block initially formed at 80 °C is amorphous but it can slowly crystallize over time after cooling to 20 °C. This leads to CDSA, which results in the gradual evolution of semiflexible rods when aging PVL_24_-PDMAC_30_ nanoparticles for 19 weeks at 20 °C or the formation of so-called ‘supermicelles’ when aging PVL_36_-PDMAC_47_ nanoparticles under the same conditions. XRD studies confirm that the initial nanoparticles have amorphous cores in each case, whereas the aged diblock copolymer nanoparticles possess mean degrees of crystallinity of 11–12% after 19 weeks. Finally, SIPLI and SAXS studies are consistent with the semiflexible rod and ‘supermicelle’ morphologies indicated by the corresponding TEM analysis. It is perhaps worth emphasizing that the *reverse sequence* aqueous PISA formulations reported herein would not require registration of any new chemical entities via REACH. This is potentially a significant cost advantage compared to alternative routes based on the statistical copolymerization of cyclic comonomers, such as CKAs or DOT.

## Supplementary Material



## Notes

Experimental details, including materials, synthetic protocols, instrumentation, analytical protocols, and additional NMR, GPC, DSC, and SIPLI (PDF)
