# Supplementary material for: Synthesis of Poly(δ-valerolactone)-poly(N,N-dimethylacrylamide) Supermicelles in Concentrated Aqueous Media via Reverse Sequence Polymerization-Induced Self-Assembly
Source: Macromolecules. 2026 Apr 3;59(7):4571–9. doi: 10.1021/acs.macromol.6c00704 (PMC13085805; doi:10.1021/acs.macromol.6c00704)
Supplement: Supplementary file 1 [file ma6c00704_si_001.pdf]

Supporting Information for:

Synthesis of poly(δ-valerolactone)-poly(N,N-dimethylacrylamide) supermicelles in concentrated aqueous media via reverse sequence polymerization-induced self-assembly

Matthew A. H. Farmer,[a] Oleksandr O. Mykhaylyk,[a] Osama M. Musa[b] and Steven P. Armes[a]

a. Dainton Building, School of Mathematics and Physical Sciences, University of Sheffield, Brook Hill, Sheffield, South Yorkshire, S3 7HF, UK.

c. Ashland Specialty Ingredients, 1005 US 202/206, Bridgewater, New Jersey, 08807, USA.

Contents

Experimental S2
  Materials S2
  Characterization Techniques S2
  Synthetic Protocols S4
Additional Characterization Data S7
References S10

Experimental

Materials

All reagents were used as received, unless stated otherwise. 4,4′-Azobis(4-cyanopentanoic acid) (ACVA; 98%), *N,N*-dicyclohexylcarbodiimide (DCC; 99%), *N*,*N*-dimethylacrylamide (DMAC; 99%), anhydrous magnesium sulfate, lithium bromide, 1,5,7-Triazabicyclo[4.4.0]dec-5-ene (TBD), δ-valerolactone and benzyl alcohol were purchased from Sigma-Aldrich (Dorset, UK). 4-(dimethylamino)pyridine (DMAP) was purchased from Alfa Aesar (Heysham, UK). DMF was purchased from VWR (Leicestershire, UK). Methanol was purchased from Fisher Scientific (Loughborough, UK). Deuterated dichloromethane (99.8%) was purchased from Goss Scientific Instruments Ltd. (Cheshire, UK). Anhydrous dichloromethane was obtained from an in-house Grubbs purification solvent system. 4-Cyano-4-(ethylsulfanylthiocarbonyl)sulfanylpentanoic acid (CEPA).[1] Deionized water was dispensed from an Elgastat Option 3A water purification system with a resistivity of 15 MΩ cm.

Characterization Techniques

^{1}H Nuclear Magnetic Resonance Spectroscopy. Spectra were recorded using a 400 MHz Bruker Avance-400 spectrometer operating at 298 K with 16 scans being averaged per spectrum. Samples were dissolved in CD_2Cl_2 and aqueous copolymer dispersions were dried with anhydrous magnesium sulfate before passing through a 0.20 µm filter. DMAC conversions were calculated by comparing the integrated vinyl proton signals at 6.63, 6.23 and 5.66 ppm with the 4.1 ppm signal assigned to the COO-C$\underline{H}_2$ ester protons on the PVL block.

Gel Permeation Chromatography. An Agilent 1260 Infinity GPC system equipped with a differential refractive index detector and a UV detector set at 305 nm was used to determine the number-average molecular weight (M_n), weight-average molecular weight (M_w) and dispersity (M_w/M_n) for each (co)polymer. Two Agilent PL-gel 5 µm Mixed-C columns and a guard column were connected in series to this GPC instrument. HPLC-grade DMF containing 10 mM LiBr was used as the eluent. GPC analysis was performed at 60 °C using a constant flow rate of 1.0 mL min^{-1}. A series of twelve near-monodisperse poly(methyl methacrylate) calibration standards with M_p values (ranging from 800 g mol^{-1} to 2 200 000 g mol^{-1}) was used to calculate molecular weights and dispersities. All (co)polymer samples were diluted to 1.0% w/w using DMF and chromatograms were analyzed using Agilent GPC/SEC software.

Differential Scanning Calorimetry (DSC). Measurements were performed using a TA DSC25 Discovery series instrument operating from –90 ºC to 200 ºC at a heating/cooling rate of 10 ºC min^{-1} using aluminum Tzero pans and standard lids. Instrument calibration was performed using an indium standard. All DSC analyses involved three heating/cooling cycles.

Transmission Electron Microscopy (TEM). Copper grids (Agar Scientific, UK) were coated in-house with a thin film of amorphous carbon and then treated with a plasma glow discharge for 30 seconds to generate a hydrophilic surface. An 8 μL droplet of freshly diluted 1.0% w/w aqueous copolymer dispersion was placed on a hydrophilic grid for 1 min, then blotted to remove excess sample. Each grid was negatively stained for a further 30 seconds using an 8 μL droplet of 0.75% w/v aqueous uranyl formate solution, which was then carefully blotted to remove excess stain. Each grid was dried with the aid of a vacuum hose. Imaging was performed using a JEOL JEM120i microscope equipped with a JEOL SightSKY™ EM-04500SKY camera operating at 80 kV.

X-Ray Diffraction (XRD). Prior to XRD analysis, all aqueous dispersions were freeze-dried overnight. Powder XRD analysis was performed using a Bruker D8 ADVANCE X-ray powder diffractometer (Cu-Kα radiation) equipped with a motorized divergence slit for Bragg-Brentano geometry and a high-resolution energy-dispersive Lynxeye XE detector. The mean degree of crystallinity, D_c, for the PVL_x-TTC precursors and the PVL_x-$PDMAC_y$ diblock copolymers was calculated using diffrac.eva v6.1 software.

Shear-induced polarized light imaging (SIPLI). Shear alignment experiments were conducted using an Anton Paar Physica MCR301 rheometer equipped with a SIPLI attachment. For these measurements, 25 mm polished steel and fused quartz plates were applied to comprise a plate−plate geometry with a zero gap of 1.00 mm. Temperature was maintained at 20 ºC using a Peltier system with a Peltier hood. Sample illumination was achieved using a high-intensity fiber optic white light source (150 W MI-150) supplied by Edmund Optics (York, UK). Polarized light images were recorded with the polarizer and analyzer axes crossed at 90° using a color CCD camera (Lumenera Lu165c). Shear was applied to the samples for 1 min prior to imaging.

Small-Angle X-ray Scattering Studies (SAXS). SAXS experiments were conducted on a 1.0% w/w aqueous dispersion of PVL_{24}–$PDMAC_{30}$ rods and the solid PVL_{24}-TTC precursor at Diamond Light Source (station I-22, Didcot, UK)[2] using monochromatic X-ray radiation [λ = 1.24 Å; q range = 0.002 to 0.239 Å^{-1}, where q is the length of the scattering vector and θ is one-half of the scattering angle, such that $q = (4\pi/\lambda).\sin\theta$] and a 2D Pilatus 2M hybrid pixel detector (Dectris, Switzerland). A flow-through glass capillary of 1.54 mm diameter was used as a sample holder. Ten SAXS patterns were recorded the sample, which were then averaged prior to analysis.

SAXS experiments were also conducted on a 1% w/w aqueous dispersion of PVL_{36}–$PDMAC_{47}$ supermicelles at the ESRF (station ID02, Grenoble, France)[3] using monochromatic X-ray radiation [λ = 1.03 Å; q range = 0.001 to 0.122 Å^{-1}] and a Eiger2 4M hybrid pixel two-dimensional (2D) detector (Dectris, Switzerland). A flow-through glass capillary of 1.89 mm diameter was used as a sample holder. Again, ten SAXS patterns were recorded the sample, which were then averaged prior to analysis.

Scattering data were reduced using standard routines provided by the beamline,[4] and were further analyzed using Irena SAS macros for Igor Pro.[5]

X-Ray Diffraction (XRD). Prior to XRD analysis, all aqueous dispersions were freeze-dried overnight. Powder XRD analysis was performed using a Bruker D8 ADVANCE X-ray powder diffractometer (Cu-Kα radiation) equipped with a motorized divergence slit for Bragg-Brentano geometry and a high-resolution energy-dispersive Lynxeye XE detector. The mean degree of crystallinity, D_c, for the PVL_{24}-TTC precursor and several PVL_{24}-$PDMAC_x$ diblock copolymers was calculated using diffrac.eva v6.1 software.

Synthetic Protocols

Synthesis of monohydroxy-capped poly(δ-Valerolactone)

The polymerization of δ-valerolactone was performed following a synthesis protocol from the literature.[6] In a flame-dried Schlenk flask, δ-valerolactone (5.00 g, 49.9 mmol) was combined with TBD (2.09 g, 15.0 mmol), benzyl alcohol (0.18 g, 1.67 mmol; target DP = 30), and toluene (35 mL). This mixture was stirred at 20 °C under a nitrogen atmosphere, and the resulting ring-opening polymerization was quenched after 15 min by precipitating into excess ice-cold methanol and filtered under vacuum, yielding a monohydroxy-capped poly(δ-valerolactone) with a mean DP of 24 (PVL_{24}-OH). This mean degree of polymerization was estimated by end-group analysis using ^{1}H NMR spectroscopy by comparing the methylene signals of the benzyl end-

group at 5.13 ppm with the PVL backbone signals at 4.08 and 3.65 ppm (see **Figure S1**). An analogous synthesis protocol was used to prepare PVL_{36}-OH where reagent quantities were adjusted to target a DP of 47 and the reaction was allowed to proceed for 1 h prior to quenching (see **Figure S2**).

Synthesis of monofunctional trithiocarbonate-capped poly(δ-Valerolactone)

All glassware was dried in a 200 °C oven for 24 h prior to use. DMAP (0.027 g, 0.22 mmol), DCC (0.84 g, 4.05 mol), monohydroxy-capped PVL_{24}-OH (3.00 g, 1.19 mmol) and CEPA (0.53 g, 2.00 mmol) were weighed into four separate dry 28 mL vials, sealed with rubber septa, and further dried in a vacuum oven for 2 h at 35 °C. A minimal amount of anhydrous CH_2Cl_2 was added to these four vials using a syringe/needle to dissolve each reagent. The three CH_2Cl_2 solutions containing CEPA, PVL_{24} and DMAP respectively were then transferred via syringe/needle into a 100 mL two-necked round-bottom flask fitted with a condenser, charged with a magnetic stirrer bar, and sealed with a rubber septum. This flask was then immersed in an ice bath and the CH_2Cl_2 solution containing DCC was added dropwise via syringe/needle. The reaction mixture was heated to reflux while purging with dry N_2 gas and then refluxed for 48 h. The reaction mixture was cooled to 20 °C, filtered to remove the insoluble *N,N*-dicyclohexylurea by-product, and the solution was concentrated to approximately 2 mL under vacuum. The crude PVL_{24}-TTC was purified by precipitation into excess ice-cold methanol, filtered under vacuum and washed copiously with ice cold methanol to remove impurities. GPC analysis (using a UV detector set at 305 nm) confirmed that the purified PVL_{24}-TTC precursor contained no residual CEPA (see **Figure S5**). ^{1}H NMR spectroscopy was used to assess the mean degree of esterification via end-group analysis, comparing the integrated proton signal at 1.91 ppm assigned to the methyl group of the RAFT agent with the unique PVL backbone signals at 4.08 ppm (see **Figure S3**). For analogous syntheses in which the mean DP of the monofunctional PVL precursor was 36, reagents were adjusted accordingly (see **Figure S4**).

Synthesis of PVL_{24}-$PDMAC_{30}$ nanoparticles by RAFT polymerization at 80 °C

A 14 mL glass vial was loaded with PVL_{24}-TTC (0.50 g, 0.18 mmol), DMAC (0.54 g, 5.41 mmol, target DP = 30), ACVA (10.1 mg, 36.0 µmol, [TTC]:[ACVA] molar ratio = 5.0) and a magnetic stirrer bar, then sealed with a rubber septum. The vial was purged with dry nitrogen gas for 30 min and then immersed in an oil bath set at 80 °C. The reaction mixture was stirred magnetically and monitored visually. When the mixture became significantly more viscous after 16 min, additional deoxygenated deionized water (2.44 mL, preheated to 80

°C, targeting 30% w/w solids) was introduced using a degassed syringe and needle. The glass vial was then removed from the oil bath, vortex-mixed for 2 min to ensure homogeneity, and returned to the oil bath. At this stage, the reaction mixture was sampled for ^{1}H NMR spectroscopy analysis, which revealed an immediate DMAC conversion of 37% (PDMAC DP ~ 11). The DMAC polymerization was allowed to continue for 16 h before being quenched by exposing the reaction mixture to air while cooling to 20 °C. ^{1}H NMR studies indicated a final DMAC conversion of more than 99%.

Synthesis of PVL_{36}-$PDMAC_{47}$ nanoparticles by RAFT polymerization at 80 °C

A 14 mL glass vial was loaded with PVL_{36}-TTC (0.10 g, 0.025 mmol), DMAC (0.012 g, 1.17 mmol, target DP = 47), ACVA (1.41 mg, 5.03 μmol, [TTC]:[ACVA] molar ratio = 5.0) and a magnetic stirrer bar, then sealed with a rubber septum. The vial was purged with dry nitrogen gas for 30 min and then immersed in an oil bath set at 80 °C. The reaction mixture was stirred magnetically and monitored visually. When the mixture became significantly more viscous after 11 min, additional deoxygenated deionized water (0.47 mL, preheated to 80 °C, targeting 30% w/w solids) was introduced using a degassed syringe and needle. The glass vial was then removed from the oil bath, vortex-mixed for 2 min to ensure homogeneity, and returned to the oil bath. At this stage, the reaction mixture was sampled for ^{1}H NMR spectroscopy analysis, which revealed an immediate DMAC conversion of 42% (PDMAC DP ~ 17). The DMAC polymerization was allowed to continue for 16 h before being quenched by exposing the reaction mixture to air while cooling to 20 °C. ^{1}H NMR studies indicated a final DMAC conversion of more than 99%.

Additional Characterization Data

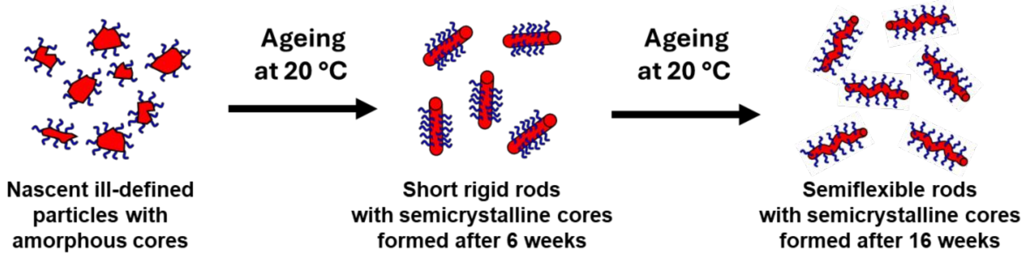

Scheme S1. Schematic representation of the morphological evolution of PVL_{24}-$PDMAC_{30}$ diblock copolymer nanoparticles during ageing at 20 °C.

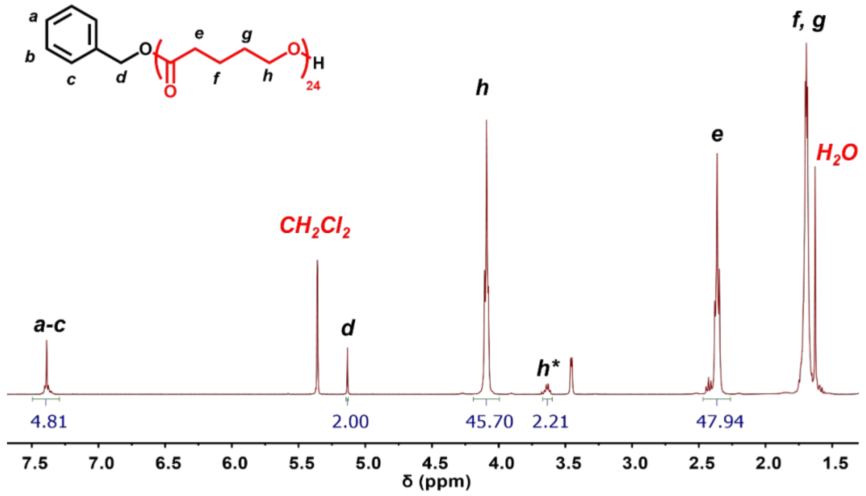

Figure S1. Assigned ^{1}H NMR spectrum (CD_2Cl_2) recorded for the PVL_{24}-OH precursor, which was prepared via TBD-catalyzed anionic ring-opening polymerization of ε-caprolactone using benzyl alcohol as an initiator (where h* represents the terminal h protons).

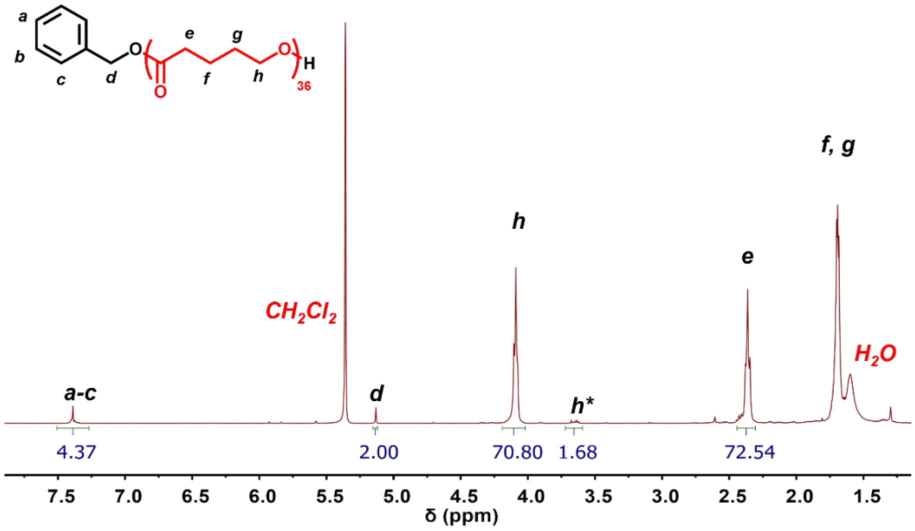

Figure S2. Assigned ^{1}H NMR spectrum (CD_2Cl_2) recorded for the PVL_{36}-OH precursor, which was prepared via TBD-catalyzed anionic ring-opening polymerization of ε-caprolactone using benzyl alcohol as an initiator (where h* represents the terminal h protons).

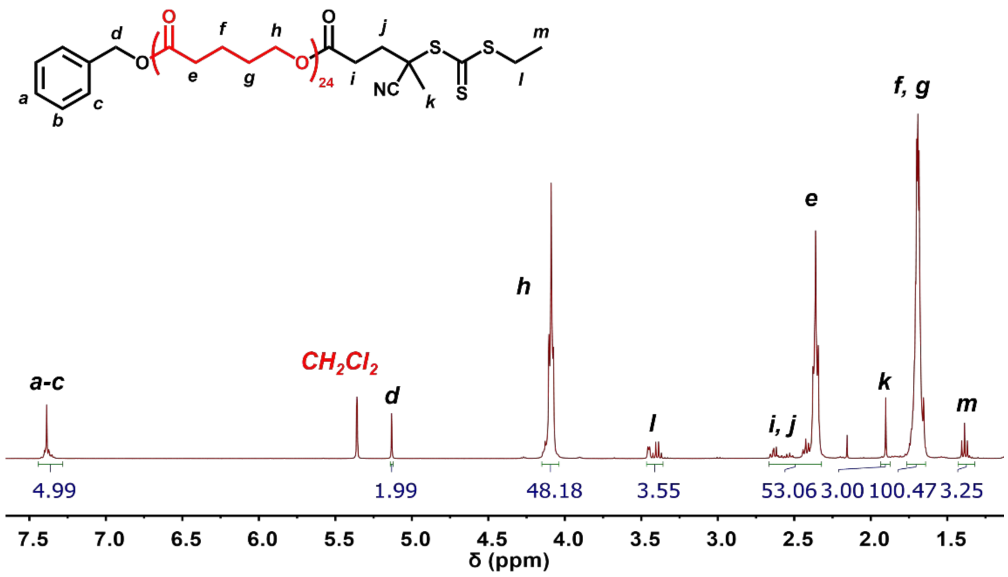

Figure S3. Assigned ^1H NMR spectrum (CD_2Cl_2) recorded for a PVL_{24}-TTC precursor, which was prepared by DCC/DMAP-catalyzed esterification of a hydroxy-capped PVL_{24} precursor using an excess of a carboxylic acid-functionalized RAFT agent (CEPA).

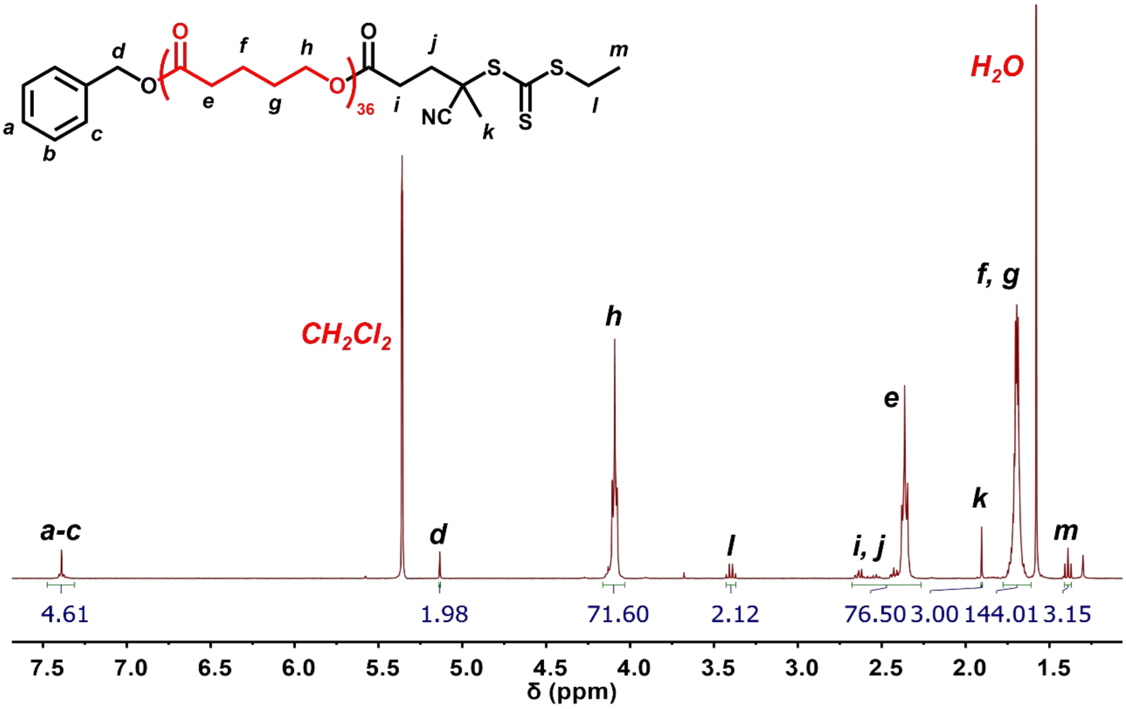

Figure S4. Assigned ^1H NMR spectrum (CD_2Cl_2) recorded for a PVL_{36}-TTC precursor, which was prepared by DCC/DMAP-catalyzed esterification of a hydroxy-capped PVL_{24} precursor using an excess of a carboxylic acid-functionalized RAFT agent (CEPA).

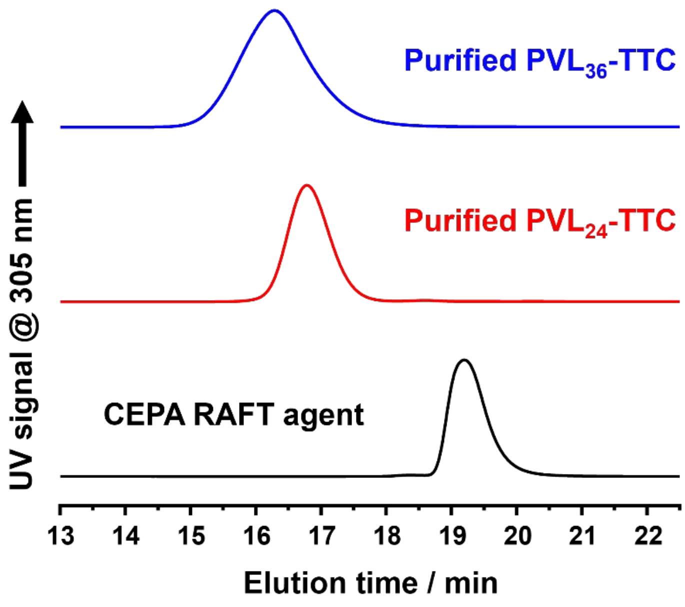

Figure S5. DMF UV GPC curves (λ = 305 nm) recorded for PVL_{24}-TTC (red curve), PVL_{36}-TTC (blue curve) and the RAFT agent CEPA (black curve). For GPC analysis of the RAFT agent, the DMF eluent contained 1.0% glacial acetic acid but no LiBr. For GPC analysis of PVL_x-TTC, the DMF eluent contained 10 mM LiBr but no glacial acetic acid.

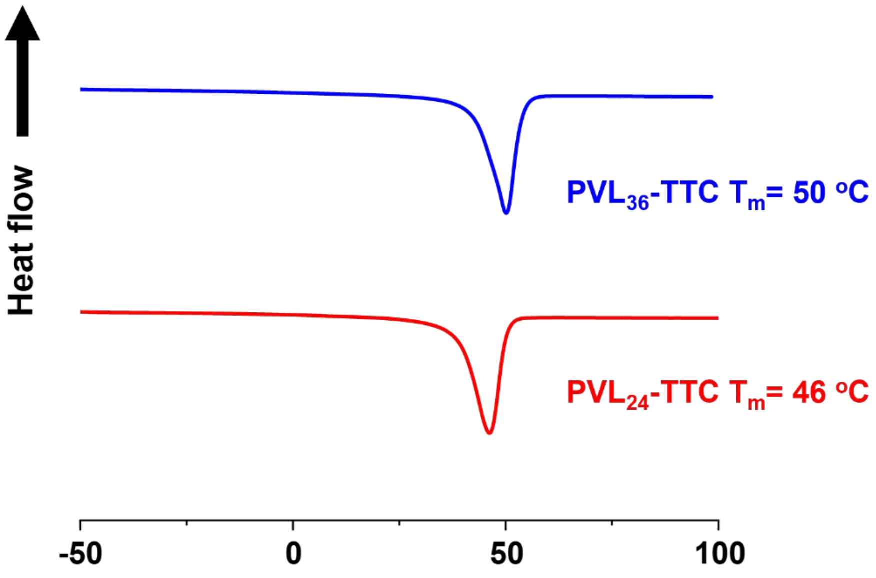

Figure S6. DSC curve recorded for the PVL_{24}-TTC precursor (red curve; T_m = 46 °C) and the PVL_{36}-TTC precursor (blue curve; T_m = 50 °C) at a heating rate of 10 °C min^{-1}.

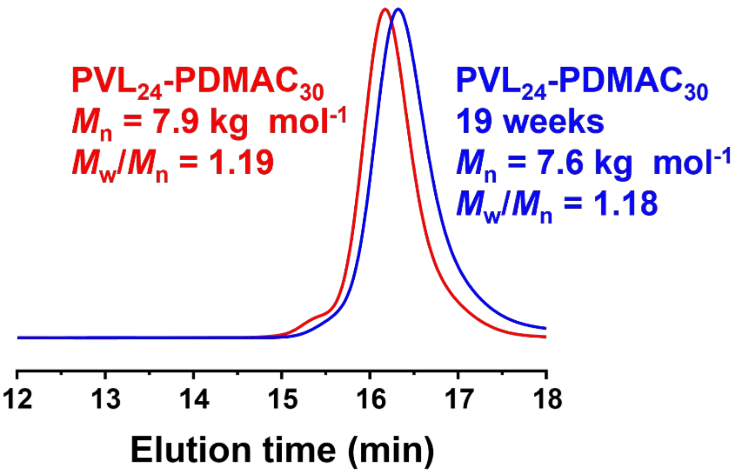

Figure S7. DMF GPC curves (refractive index detector) recorded for the PVL_{24}-$PDMAC_{30}$ diblock copolymer immediately after synthesis (red curve), and the same polymer after 19 weeks of ageing at 20 ºC (blue curve).

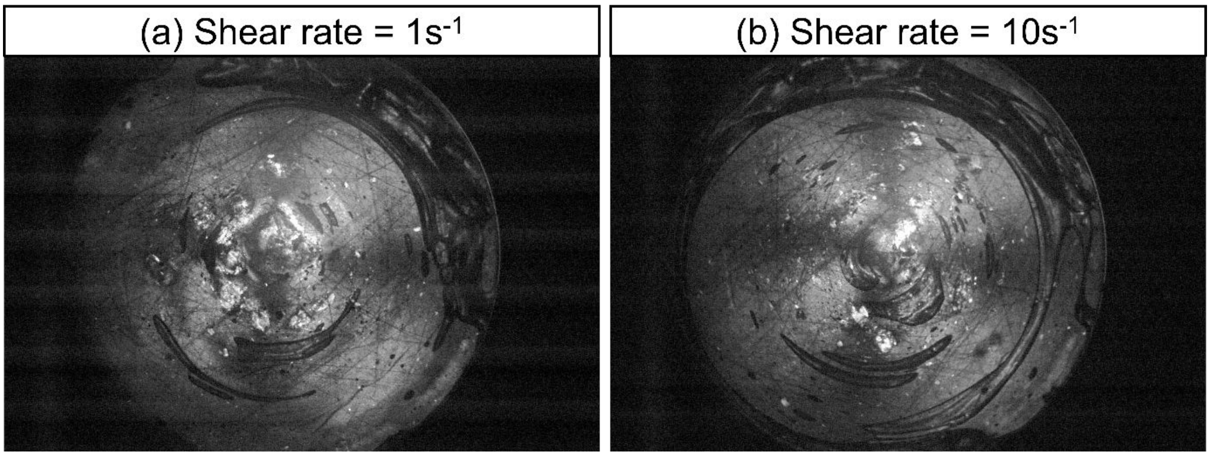

Figure S8. Digital photographs recorded at 20 °C for a 30% w/w aqueous dispersion of PVL_{24}-$PDMAC_{30}$ nanoparticles at an applied maximum shear rate of (a) 1 s^{-1} and (b) 10 s^{-1}. The distinctive Maltese cross indicates shear alignment of the semiflexible rods under such conditions. The original image was converted into grayscale for clarity.

References

1. Danial, M.; Telwatte, S.; Tyssen, D.; Cosson, S.; Tachedjian, G.; Moad, G.; Postma, A. Combination Anti-HIV Therapy via Tandem Release of Prodrugs from Macromolecular Carriers. *Polym. Chem.* **2016**, *7*, 7477–7487.
2. Smith, A. J.; Alcock, S. G.; Davidson, L. S.; Emmins, J. H.; Hiller Bardsley, J. C.;

Holloway, P.; Malfois, M.; Marshall, A. R.; Pizzey, C. L.; Rogers, S. E.; Shebanova, O.; Snow, T.; Sutter, J. P.; Williams, E. P.; Terrill, N. I22: SAXS/WAXS Beamline at Diamond Light Source – An Overview of 10 Years Operation. J. *J. Synchrotron Radiat.* **2021**, *28*, 939–947.

3. Narayanan, T.; Sztucki, M.; Zinn, T.; Kieffer, J.; Homs-Puron, A.; Gorini, J.; Van Vaerenbergh, P.; Boesecke, P. Performance of the Time-Resolved Ultra-Small-Angle X-Ray Scattering Beamline with the Extremely Brilliant Source. *J. Appl. Crystallogr.* **2022**, *55*, 98–111.
4. Filik, J.; Ashton, A. W.; Chang, P. C. Y.; Chater, P. A.; Day, S. J.; Drakopoulos, M.; Gerring, M. W.; Hart, M. L.; Magdysyuk, O. V.; Michalik, S.; Smith, A.; Tang, C. C.; Terrill, N. J.; Wharmby, M. T.; Wilhelm, H. Processing Two-Dimensional X-Ray Diffraction and Small-Angle Scattering Data in DAWN 2. *J. Appl. Crystallogr.* **2017**, *50*, 959–966.
5. Ilavsky, J.; Jemian, P. R. Irena: Tool Suite for Modeling and Analysis of Small-Angle Scattering. *J. Appl. Crystallogr.* **2009**, *42*, 347–353.
6. Lohmeijer, B. G. G.; Pratt, R. C.; Leibfarth, F.; Logan, J. W.; Long, D. A.; Dove, A. P.; Nederberg, F.; Choi, J.; Wade, C.; Waymouth, R. M.; Hedrick, J. L. Guanidine and Amidine Organocatalysts for Ring-Opening Polymerization of Cyclic Esters. *Macromolecules* **2006**, *39*, 8574–8583.
